# Co-creation as an innovative setting to improve the uptake of scientific knowledge: overcoming obstacles, understanding considerations and applying enablers to improve scientific impact in society

**DOI:** 10.1186/s13731-021-00176-2

**Published:** 2021-09-26

**Authors:** J. Stier, S. E. Smit

**Affiliations:** 1 Mälardalen University, Västerås, Sweden; 2 Groningen, The Netherlands

**Keywords:** Scientific knowledge, Societal impact, Co-creation, Quadruple helix, Societal challenges

## Abstract

Impact-driven research is a EU priority and, increasingly, for universities around Europe. Still, there is need for specific strategies to improve the societal impact of scientific knowledge and therewith improve the uptake of scientific results. Co-creation deeply evolves the role of scientific knowledge and increases its impact. Albeit there is much research on the conceptualization and contextualization of co-creation, research on the microlevel dynamics of co-creation is less common. This article aims to understand the dynamics of and clarify the role of co-creation within and between quadruple helix actors (academia, government, industry and societal partners). Here, co-creation refers to the collaboration, where such actors actively join forces to address challenges. This paper revolves around insights from the European Commission Horizon 2020-project—*Accomplissh* (www.accomplissh.eu) which stands for “Accelerate co-creation by setting up a multi-actor platform for impact from Social Sciences and Humanities”. The results lay bare a set of obstacles, areas of consideration and enablers in co-creation. This said, it is argued that scientific knowledge is optimally utilized when a set of guidelines or recommendations are followed and carried out by all involved actors.

## Introduction

Historically the university institution is strongly connected to society; the word university is derived from the Latin word *Universitas* meaning “the whole, the universe, the world”. However, whether universities get engaged in society, has become a strategic management choice in the central mission of universities. As Grau et al. (2017) argue: universities are either part of the problem—by neglecting regional, national or global commitments, or part of the solution—by acting in favor of the public good. Universities of the latter “type” have contributed with scientific knowledge to society to meet societal challenges and bring new insights to the (policy) tables.

The last decade, the focus on higher education and research as key players in solving the “grand challenges” identified in Agenda 2030 has been accentuated (see, for instance, Berkman, 2020; International Science Council, 2019; Stier, 2020). It is also in this light that Commissioner Carlos Moedas in 2015 declaration of the necessity of an open society, open innovation and open science should be seen. After all, the high level of complexity of society’s current day challenges requires a multidisciplinary approach. This being said, European universities are encouraged or obliged to initiate impact-driven research and co-creation with actors outside academia. This goes beyond the more traditional view on science communication often referred to as knowledge transfer or knowledge valorization. Today, therefore, we talk about societal impact *from* science, peppered with terms such as co-creation, impact-driven research, Science 2.0, citizen science, public engagement and open science, including open access, and open data. Similarly, the last decade increasing attention has been drawn to knowledge exchange, co-creation, science-informed policy-making and the overall role of academia for societal development (Gluckman, 2018; International Science Council, 2019; Oliver & Cairny, 2019; Phipps & Morton, 2013).

In recent years has often argued that academia, government, industry and societal partners (four sectors commonly summarized with the concept of quadruple helix) have much to gain from intersectoral collaboration (Johnston, 2018; Stier & Axelson, 2020). From a policy-making perspective such collaboration is viewed increasingly as a necessity when puzzled with the grand challenges—may it be economic recessions, public health concerns—like a pandemic, global warming or trying to accommodate austerity or counteract extremism.

For these reasons, co-creation managers, innovation officers or impact champions do their best to convince researchers of the value of co-creation. These ambitions notwithstanding, universities and their managements often remain ambivalent—at least beyond mission statements, policies and public rhetoric to these changes. For researchers, this course change is not without challenges. As Laing et al. (2017, p. 13) write: “Academics do not operate in isolation from their institutional context, and activity is guided day to day by the policies and procedures adopted by their universities, in turn guided by national policies and international frameworks.” (see Upton et al., 2014).

Higher education and research bring forward an immense number of scientific publications with valuable knowledge and insights for—among others—policy-makers and politicians to sharpen their policy proposals and opinions. Yet, it does not come naturally for scientific knowledge to find its way into wider set of possible end-users. To accommodate this, there are numerous concepts depicting scientific outcomes, connected to a wider range of actors beyond the academic community and scientific debates—such as “knowledge mobilization”, “knowledge communication” and “knowledge valorization”.

“Knowledge mobilization” refers to the process of understanding the added value of scientific knowledge in the impact process, and in particular in using co-creation as a model (Bannister & Hardill, 2013). This said, it is important to distinguish between different forms of knowledge mobilization processes (see Phipps & Cummings, 2016).

“Knowledge communication” through mainstream media and scientific publications, pertains to knowledge dissemination, whereas “knowledge translation” steps up and translates scientific outcomes for wider purpose, beyond the academic field, to ensure practical use. This differs from “knowledge transfer” i.e., sharing knowledge from one field to another.^1^ “Knowledge translation” is close to knowledge exchange, when data and results from research are transformed to be of directly benefit for the wider public. All these different knowledge mobilization processes can lead to scientific impact.

Moreover, “knowledge valorization” is often referred to the creation of value *from* academic knowledge by making knowledge *available* beyond the academic field. Bayley and Phipps (2018) state that advocates of societal impact are seeking the same value creation. However, the art of societal impact creation goes beyond making knowledge available; it requires a set of skills and insights to be able to understand, appraise and make decisions about how to connect the scientific knowledge to the outside world. In the next step, it also entails joint knowledge production—co-creation of knowledge.

Against this background, it is clear that there is a highly diverse discourse on co-creation. The same observation applies for research in the area of co-creation—which is ample and diverse.

## Previous research

Grønvad et al. (2017) conclude that in policy reports and academic articles co-creation is given two meanings.^2^ One is the co-creation of public policies, the other the co-creation of research and innovation.

Co-creation is also a key concern in a number of academic and non-academic fields. Greenhalgh et al. (2016) discern four fields: business and management studies (“value co‐creation”—e. g. Alves et al., 2016; Ramaswamy & Ozcan, 2018), design science (“experience‐based co‐design”), computer science (“technology co‐design”), and community development (“participatory research”). In addition to these fields, there is research on co-creation in science and technology studies (Jasanoff, 2004), quadruple helix collaboration in forestry (Grundel & Dahlström, 2016), fisheries andm marine resources (Runnebaum et al., 2019), energy renewal (García-Terán & Skoglund, 2019), journalism (Heikka & Carayannis, 2019), consumer participation (Siverstøl, 2018) and in the health and welfare field (Holmström et al., 2016; Needham, 2008). Much research also revolves around conceptual issues, policy, valorization and rhetorics (Greenhalgh et al., 2016; Voorberg et al., 2015). In addition, there is research on the importance of intermediaries and knowledge brokers (2009b; Bornbaum et al., 2015; Ward et al., 2009a).

In the scientific literature, co-creation has still a strong connotation to the economic disciplines. It is viewed as a result of the emphasis on value. Co-creation is often considered as a more general concept encompassing the specific theoretical and empirical occurrences in which companies and customers generate value through interaction (Vargo & Lusch, 2008).

In our case, co-creation refers to the collaboration between a variety of actors actively joining forces to tackle jointly defined challenges (see, for instance, Jasanoff, 2004). Actors might belong to various sectors of society, often between the previous mentioned quadruple helix: academia, government, industry and societal partners. Shared expertise and experience from the helix actors lead to both the identification of joint challenges and to innovative insights and solutions even. These outcomes would not be realized should all actors try and tackle the issues at hand as individual actors.

As it was shown, there is much research on the conceptualization and contextualization of co-creation, whereas there is less research on the microlevel dynamics involving representatives from academia, civil society, government and industry and shedding light upon on relation-building, mutual respect, trust or confidence, or for that matter, on its challenges, dilemmas or on potential enablers. To fill this research gap was one of the objectives of the European Commission Horizon 2020 project—*Accomplissh*^3^ (www.accomplissh.eu).

## Aim of paper and methodological design

The acronym Accomplissh stands for “Accelerate co-creation by setting up a multi-actor platform for impact from Social Sciences and Humanities”. The overall aim of the project was to strengthen the position and impact generation of social sciences and humanities research and contribute to societal impact through dialogue between academia, government, industry and societal partners. An important element in the research was to involve stakeholders from all quadruple helix partners and make use of their experiences and best practice examples. In the first year of the project, an integral part of reaching this objective, therefore, was to analyze co-creation in theory and practice. In doing this, these partners, jointly and equally, were to identify barriers and enablers of co-creation, with the goal of initiating, widening and optimizing co-creation.

Based on the focus group interviews this paper potential barriers and enablers of co-creation. The goal is to fill the research gap on the microlevel dynamics of co-creation. Another goal is to increase the knowledge of the “nuts and bolts” of co-creation—i.e., to offer a set of tools facilitating co-creation.

The focus group interviews were led by a facilitator and guided by an interview guide. In total, 14 focus group interviews were conducted with people from academia, civil society, industry, and government in 12 European countries. Interviews were conducted in the national language of the participants. Interview participants were selected using purposive sampling—i.e., participants were identified and approached due to their roles in and experiences of co-creation. Those willing to participate were included in the final sample. All in all, there were 33 interview participants from academia, 23 from civil society, 11 from industry and 18 from government.

At the start of the interviews, the participants were first informed about the project’s objectives and setup of the focus group interviews. Then, they were inquired about their experiences and lessons learned from collaboration between actors from academia and other sectors of society.^4^ They were also asked to single out and discuss 5 to 7 obstacles and 5 to 7 enablers in such collaboration setting.

Interviews were transcribed and then translated into English, and after that subject to a qualitative analysis by one of the authors of this text and a colleague of his. In the analysis, obstacles to and enablers of co-creation were identified.

## Results and discussion

In the focus groups interviews, the participants from academia, civil society, industry and government describe a number of obstacles and enablers. Let us take a close look at these.

### The problem of problems

It may go without saying—but collaboration begins with a common concern or a problem of some sort. Yet, many of the focus groups participants have experiences of misunderstandings emanating from different and ambiguous understandings of what a problem is, due to the fact that such a problem is conceptualized within the realms of different professional contexts (see Gluckman, 2018). One academic partner says: “[T]here are those projects that quite easily defined. However, most cases where it’s about bringing business and academia together are not quite as straightforward… //.. Because there is no scientifically definitive solution to an economic problem, but rather there is scientific input, there are opportunities for information and education.”

Also, even if the same word or term is used to describe such a concern or problem it may be seen very differently by those involved. For example, what in the initial stages of a collaboration is referred to as a social problem by the involved parties, may be a very different thing. Gluckman (2018, p. 96) writes: “We are very good at problem definition” (p. 96). From a sociology perspective, a social problem is not necessarily seen in the same way as it is for a street level social worker. By the same token, a problem to society is not automatically a scientific problem.

Therefore, the focus groups participants argue that when quadruple helix partners come together with a common interest (for instance alcohol abuse) they need to define the problem together—to find a common epistemological ground, once again, to refer to Gluckman—so that the outcome of the collaboration both is consistent with the objectives of academia and the other parties (in our case, that alcohol abuse is analyzed as a research problem, while the results assist, for instance a municipality, to come to terms with young alcohol abusers). The focus groups participants view the joint problem formulation phase as crucial and stress the need for setting aside sufficient time for this collaboration phase. This viewpoint is also consistent with the findings of Stier and Axelson (2020). It becomes motivational the baseline from which the collaboration starts—and is imperative for a smooth ride together.

### Different languages, terminology and communication

Based on what was just said, quadruple helix partners speak different languages and ascribe different connotations to terms and concepts (Holmström et al., 2016; Axelson & Stier, 2020; Bergfeld et al., 2021). For the focus groups participants there is a great deal of conceptual confusion out there, around terms such as public engagement, co-creation, valorization and impact—to mention a few. These terms are used frequently without being properly defined, nor questioned. In the focus group interviews, an industry partner vividly describes an obstacle in the collaboration with academia:

I think a big problem I have to deal with is the confusion of tongues. The language academics use is so different from the language used by corporate leaders, and hugely different from the languages used by creatives. We don’t understand each other.//:: we have to understand each other before we [collaborate].

The participants provide numerous accounts about miscommunication; how some concepts are ambiguous or unfamiliar; how the involved parties speak the same language but mean different things; or how words are assumed to carry the same meaning and emotional valor, which eventually lead to misunderstandings or communication breakdowns. As we saw, when academics refer to a problem they typically—and unreflexively—mean *research* problem, whereas a civil servant may assume it is a *policy* problem or a *health* problem. Or going back to a previous example; the word ‘social’ means different things. For a sociologist it is juxtaposed with the individual, for a civil servant it may connote with the social services. In addition, at a dinner, a *social* gathering means eating a meal, having a glass of wine and *socializing*. Or another example; the word critical. As social scientists we claim to be critical and by this we refer to a specific stance towards things. It is an approach or a gaze, whereas for the majority of people critical equals have a negative attitude towards somebody or someone.

This said, the focus group participants agree that when working together it is essential to define the meaning of concepts and nomenclature as soon as possible in the collaboration process. It is also imperative to discuss the meanings and emotional loading of words and terms. In addition, more importantly, to recognize the fact that the involved parties often take such meanings and connotation for granted—at least until they misunderstand one other. Academics must better communicate what we mean when we say things and, as much as possible refrain from ambiguous communication and professional jargon. This also applies to international collaboration. I hear British colleagues talk with their international partners about the National Health Service and assume that the rest of us know what they are talking about, whereas their Swedish colleagues expect other to have knowledge about the Swedish research funding system. Therefore, such blind spots in communication needs to be addressed otherwise unnecessary misunderstanding occur.

Furthermore, the focus group participants stress the fact that the rhythm and sequence of communication differ between academia and many other domains of society. Often, academic communication come across as winded and unnecessarily complicated. In the eyes of others, the guise of communication sometimes seems more important than the message of the communication. Thus, the *what* yields to the *how* when we talk with our collaboration partners, which at times creates an unnecessary distance between us. In the initial phases of collaboration with people outside academia it is, therefore, imperative to avoid using an unnecessarily complicate language.

### Different institutional logics and motivations

In the focus groups there is widespread agreement that co-creation is crucial and worth pursuing. As one academic participant puts it: “… collaboration is not only a value; it is a necessity//.. we actually collaborate because this is necessary to achieve an aim that we wouldn’t be able to achieve alone…” Yet, people and organization alike, are driven by different incentives, objectives and rationales. An industry partner expressed this in the following way:”[S]ometimes I do blame the universities. Universities have a strong focus on the past, and a focus on output, like: The evidence. However, colleges are more focused on innovation”. Gluckman (2018, p. 93) writes: “Science and policy are very different cultures: they have distinct methods and epistemologies.” Thus, non-profit organizations, corporations, municipalities and researchers tend to have divergent views on what they do and why they do it. Making a difference, the bottom line, good service and life quality for citizens and state-of-the-art research may all be desired outcomes of quadruple helix collaboration, but these goals may be prioritized differently by those involved.

For these reasons, the focus group participants emphasize the need for early stage communication among collaboration partners, including spelling out questions such as: what are your goals?; what are your reasons for doing this?; what are the foreseeable and desirable outcomes?; and, what are the deliverables, foreseeable results and potential impact? Academia, civil society, government and industry are bound by particular legal frameworks and value systems, have dissimilar missions for the work and also become involved in collaboration for a variety of reasons. Therefore, there are great differences in the motivation and expectations quadruple helix actors have of such collaboration.

In addition, and as a public sector partner says: It’s about the setting of incentive systems for academia and for young academics…//.. incentives are set in such a way that [researchers] aren’t interested, can’t be interested.” Many academics do not view research as merely a tool to meet a societal challenge or a need for innovation. Doing research is motivating in itself, and scientific knowledge, stringency and rigor are ends in themselves.

As researchers, they are part of an academic rewarding system and often assessed by their scientific impact and scientific publications rather than the societal impact they have has contributed to.

In the focus groups it also becomes clear that there are dissimilar views on how to approach, work with and present collaboration outcomes. As one non-academic focus group participant puts it: “Then, suddenly, a report appears in our email, with a friendly thanks and goodbye—no intentions to help us understand or make use of the findings or conclusions”. To the non-academic partners outputs and outcomes of a joint efforts may it patents, policies, methods, or interventions. By contrast, for academics, publications and conference presentations are obvious and the most important deliverables of any research-oriented project or collaboration. The credibility of the outcomes is largely based on an international peer-review system, whereas non-academic co-creation partners base credibility of research and co-creation outputs on professional relevance or in the light of their own motivations and experiences (Lupia, 2013; Runnebaum et al., 2019).

In summary, according to the focus group participants, successful collaboration demands an open discussion on how the involved parties view the outcomes, outputs and impact of the joint efforts—both short and long-term ones. For instance, what is a project result and when can they be presented? Who owns the data and results? What conclusions can be drawn with sufficient levels of certainty and credibility? Many researchers are reluctant to present anything but the *final* results (typically after a few years of hard work), whereas, and as stated earlier, the collaboration partners prefer recurrent “control stations” and need periodic reports of preliminary results through-out the project cycle. Moreover, the focus group participants point to the fact often there is lack of capacity or lack of engagement from key people in the organizations involved, which results in a situation, where insufficient resources are allocated to the task at hand. This said, those who initiate a conversation on collaboration need to have (or organize) the organization’s mandate and access to resources.

### Different roles

Research on co-creation (Alexander, 1994; Greenhalgh et al., 2016) points to the “two cultures problem”—i.e., a situation where academics and non-academic stakeholders do not engage with or understand one another. In part, this problem emanates in the above mentioned differences in institutional logics, motivations, time horizons. These differences materialize in different *roles* of the co-creation parties (Gluckman, 2018). As an academic partner puts it:

This, I think, is a challenge because all [co-creation partners] have many roles, and it is not always easy to achieve results in the exact way you would like and at a point in time you might like.

With the variety of roles, come diverging expectations (Gluckman, 2018). For instance, researchers in the social sciences and the humanities should assent to the openness of science, at the same time as they are obliged to maintain their integrity and professionalism. Many researchers do not view economic growth, profit or resolving social problems as their responsibilities. This being said, the roles of those involved in co-creation are not always mutually clear to those collaborating, including different views of the *researcher* role (Holmström et al., 2016). Acivil society partner says:

I really think that school teachers and head teachers are not really so open to cooperate with universities in research. The researcher is seen as an enemy, a war machine, which goes into schools to assess ad administrate questionnaires.

Unclarities in roles can cause those involved in co-creation to feel as if responsibilities that are not theirs are placed onto their shoulders. This can raise ethical dilemmas. For instance, should researchers provide a for-profit organization with research results that enable them to target people with the intent to increase their consumption? What if our research is used to circumvent people’s individual rights and freedoms. Or should for-profit organization fund research that does not add to their bottom line and the share holders’ profit? And so on.

To prevent problems further down the road, the focus group participants stress the importance of being frank early in the collaboration process with what one sees as one’s role in the co-creation. In doing so, it may be necessary to convey the objectives and underpinning values of one’s work and organization. These things can be addressed, for example in preparatory workshops, where the involved parties, in a controlled fashion, are allowed to share mutual views and expectations. Doing this, serves as a bonding and dedramatizing tool and may counteract feelings of inequal power and status.

### Different time conceptions

From the focus group interviews it becomes clear that differences in institutional logics also materialize in different—and often—incompatible time logics. A focus group participant from government said: “We really do have different time frames—the university’s ‘now’ is not ‘now’ for the industry. To combine these—what would be the middle way?”.

More than a decade ago (2007, p. 83), Menzies and Newson wrote: “The ‘ivory tower’ has been breached. The university is no longer a refuge from the hustle–bustle, a slow zone for reading and reflection, critical dialogue and knowledge creation—to the extent that it ever was. However, it is not merely a change in time logics *within* academia; there is also one between academia and other co-creation partners. To people outside academia the academic way of working often comes across as slow and tardy. In the focus group interviews, it becomes clear that non-academic partners expect collaboration outputs much quicker and earlier in the process that their academic partners. To paraphrase Menzies and Newson (2007, p. 93): instrumental productivity and short-term deliverables trumps theoretical interpretation and academic reflection.

By the same token, in the focus groups a governmental agency representative says: “After the collaborative agreement is signed, we hear nothing—sometimes for a year and more”. The non-academic partners prefer collaboration outputs to be inputs that feed into their work *throughout* the collaboration, and some of them are critical to researchers who work for years, without any feedback to their stakeholders, and then hand over a final report.

Conversely, academic focus group participants feel that non-academic stakeholders underestimate the time need for conducting solid research and “prefer hastiness in front of solidness”. As one academic said: “what a company wants to resolve in 2 months, researchers see as a 3-year pilot-study”. In the focus groups, there are examples, where the people involved may be unable to set aside time for the initial discussions. They also may be (happily?) unaware of what is needed from them in terms of time and efforts or unwilling to get engaged.

If those involved fail to recognize the differences in time horizons or underestimate the amount of time needed to accommodate the needs and wishes of all partners involved, the focus group participants argue that this eventually may lead to inefficiency or breakdown in co-creation. To avoid unnecessary disappointments, friction or conflicts, the focus group interview participants, therefore, agree that it is important to be up-front with what and one expects from the collaboration and *when* one expects this. It is also crucial to arrive at a common baseline for the collaboration—i.e., to describe, motivate and discuss the differences in institutional logics and times frames in academia, civil society, industry and government. In addition, a plan including regular control points when those involved meet and update one another of the progress (or lack thereof) of the work is useful. This includes deliverables and a division of responsibilities.

### Facilitating collaboration and learning from others

Among the focus groups participants there is agreement that spaces of interaction are essential to facilitate fruitful and continuous communication throughout a collaboration. One civil society organization participant says: “When [collaboration is] not coupled with physical contact, it never gets off. Or when it gets off, it dies a slow death.” So, several of the academic participants stress the necessity of meeting regularly and preferably not only in meeting rooms at the university but in the locations of your collaboration partner or elsewhere. Apart from physical locations, digital spaces can also be helpful, as long as they are properly prepared and moderated (a lesson learned from the last year of COVID-19 lockdowns).

These control points are both opportunities for the researchers to report findings, but also for the other actors to provide valuable input to the work ahead. In addition,, not to forget, it is a way for the latter to ensure to themselves and their management that everything proceeds as planned, because in one way or the other, they are held accountable for allocating time and money for the collaboration.

Moreover, among the focus group participants, there is consensus that much is to be gained from the knowledge and experiences (good and bad) of other people and their collaborative efforts. As an academic partner puts it: “It is precisely because there is an advantage in collaborating that people must find a way to collaborate, because if they do not collaborate, they don’t achieve anything”. There are numerous expressions for the value outcomes of collaboration and co-creation, including, but not limited to “mutual learning”, “knowledge transfer”, “best practice” or “idea sharing”.

At the same time, many focus groups participants stress the need for better tools assisting oneself in *how* one learns from others. All too often, so called good examples or best practice are presented to an audience, without relating them to concrete means for optimizing practice and collaboration. Thus, more effort is needed when it comes to transforming others’ practice into ours. In this endeavor the focus group participants emphasis the important roles of intermediaries, that is, experienced people who are good pedagogues and can train others in enhancing collaboration and practice.

As it was stated earlier, there is an increasing body of research on intermediaries and knowledge brokers (2009b; Bornbaum et al., 2015; Ward et al., 2009a). In the focus groups interviews, it is claimed that intermediaries and knowledge brokers are crucial in co-creation. An academic partner says: For me it’s very important that you have good facilitators; good intermediates that can shape the conversation, so that everybody who’s part in the group can be heard…. To do the translation”.

Ideally, the knowledge broker is an open-minded non-hierarchical professional, who does not make independent decisions. A knowledge broker facilitates the process and guides group decision-making while constantly inspiring the group by asking new questions or adding new perspectives. Active participation of all participants is key, so the knowledge broker encourages silent participants to join in. He or she creates trust in the group and makes sure interactions are fair. In guiding the conversation to a higher level, the knowledge broker evaluates the process ongoing and redirects if needed. He or she synthesizes ideas and information, in such a way that the partners in the co-creation collaboration are focused on the joint outcome, rather than thinking of “what they wanted to get out of it”. For these reasons, the knowledge broker is indispensable in Quadruple Helix collaboration according to the focus group participants.

## Conclusions

Co-creation and impact-driven research is a priority for the EU and, increasingly, also for universities around Europe. This notwithstanding, surprisingly little attention has been paid by researchers to microlevel co-creation dynamics.

In the interviews conducted within the Accomplissh-project, it becomes clear that collaboration between academia and other actors is important and worthwhile pursuing. There are many interview accounts stressing how much can be learned from and accomplished by such collaboration—if collaboration is both systematic and long-term—and if the those involved are willing to constantly monitor and develop their interaction with other stakeholders. At the same time, there are many examples of when collaboration in general, and with stakeholders outside academia in particular, (or for that matter *within* academia) is not always smooth and void of dilemmas.

Co-creation actors involved in are driven by different incentives, objectives, and rationales. They have different roles and typically adhere to divergent time horizons. Therefore, when academics working with, for instance, with government, they need to understand the political reality and the policy cycle and at which stage of cycle the he or she steps in. Similarly, if they collaborate with civil society money is strictly limited, and when working with industry bottom line is focal. Conversely, non-academic partners, must understand that researchers are predominantly assessed on their scientific impact and number of scientific publications rather than the amount of societal impact or profit.

A lesson from the focus group interviews is that such differences in incentives, objectives, rationales, and roles must be communicated at the onset to all the collaboration partners. In this endeavor—and for the collaboration as a whole—the focus group participants emphasize the usefulness of knowledge brokers. Similarly, much is to be gained from learning from each other, and best practice. The need for engagement and commitment from everyone involved is also stressed. Who does what, how and when? How are results received by funding bodies and who communicates results and outcomes? These are questions to consider, as early as possible in the collaboration.

Against this background, are not the things we have discussed focal considerations in all collaborations? Yes and no. Of course, much of the things addressed here boil down to all-human matters, such as mutual trust, confidence, clarity, passion, communication, language, roles, and personal motivations—regardless of whether this is a question of joint work within or without academia or not. Yet, there is some uniquely different with the complexity with quadruple helix collaboration. Any quadruple helix co-creation starts, or never starts and ends at the interpersonal level—in the space between people involved in communication and *engaged* in efforts to reach a common goal. Appreciating this, a conclusion from the focus groups interviews is, therefore, that dialogue is the key to a successful collaboration and that knowledge brokers play a key role in quadruple helix collaboration. Another is that there are a set of crucial considerations specific to quadruple helix co-creation that should be made.

From the from the focus group interviews, eleven conclusions can be drawn. To optimize the environment for societal impact and co-creation it is essential to:

- Allocate reasonable time, sufficient financial funds and adequate human resources
- Involve all stakeholders when defining the common area of concern from the outset
- Nurture stakeholder relationships
- Address differences in institutional logic, rationale, incentives, and roles
- Address differences in nomenclature, language, and modes of communication
- Challenge one’s own and each other’s thinking
- Provide platforms and spaces for interaction
- Make use of knowledge brokers to optimize collaboration
- Learn from good practice and research
- Address questions of impact, validation and valorization from the outset
- Make the case for the social sciences and humanities.

Yet, with a microlevel focus on co-creation, these conclusions go beyond the cited research and make up a contribution to the research field as a whole. Moreover, they are focal if successful and sustainable co-creation is the goal. In addition, a truly more open, engaged and collaboration-driven research agenda must adapt itself more to the world of which it is a part, without surrendering its professionalism, integrity, academic values and unique traditions. Thus, if the goal of academia is to co-create in the true sense of the word, researchers need to alter their mode of communication according to the target group. Reversibly, politicians, tech-designers or voluntary social workers need to explain their nomenclature and adapt their modes of communication to other target groups.

There are limitations to this study. It is based on the accounts of approximately 100 people. Yet, much of their experiences are both recognizable and valuable for those involved in co-creation. Because by sharing experiences, we learn more and by talking less and listening more, much more can be achieved! This builds mutual confidence is one’s abilities, motivation and sincerity. For no matter what, research and higher education are essential elements in our endeavor to meet the challenges facing humanity today and tomorrow under the condition that we get a better grip of how to best employ scientific knowledge in co-creation processes!

## Data Availability

All research data is openly accessible through the University of Groningen, data-manager is Sharon Smit, to access data contact: researchdata@rug.nl.

## Footnotes

1 Other, related terms are”knowledge creation” and”knowledge management”.

2 This also applies to”co-production”, a term often used synonymously with co-creation—see Grønvad et al. (2017).

3 The *Accomplissh* project received funding the European Union’s Horizon 2020 research and innovation programme under Grant Agreement No 693477.

4 Stier and Dobers (2017) and Dobers and Stier (2018).
